# A Brief Educational Intervention Enhances Basic Cancer Literacy Among Kentucky Middle and High School Students

**DOI:** 10.1007/s13187-020-01696-3

**Published:** 2020-01-28

**Authors:** Lauren Hudson, Kerrigan M. Samons, Haley E. Dicken, Chris Prichard, L. Todd Weiss, Jean Edward, Robin C. Vanderpool, Nathan L. Vanderford

**Affiliations:** 1 Markey Cancer Center, University of Kentucky, Lexington, KY USA; 2 College of Nursing, University of Kentucky, Lexington, KY USA; 3 Department of Toxicology & Cancer Biology, College of Medicine, University of Kentucky, Ben F. Roach Building, 800 Rose Street, CC140, Lexington, KY 40536-0096 USA

**Keywords:** Cancer, Cancer disparities, Cancer education, Cancer literacy, Educational intervention

## Abstract

Kentucky experiences the highest overall cancer incidence and mortality rates in the USA with the greatest burden in the eastern, Appalachian region of the state. Cancer disparities in Kentucky are driven in part by poor health behaviors, poverty, lack of health care access, low education levels, and low health literacy. Individuals with inadequate health literacy are less likely to participate in preventive measures such as obtaining screenings and making healthy lifestyle choices, thus increasing their chances of developing and dying from cancer. By increasing cancer literacy among youth and adults, it may be possible to decrease cancer disparities across Kentucky. This study aimed to establish connections with middle and high schools in Kentucky that would facilitate pilot implementation of a brief cancer education intervention and assessment of cancer health literacy among these student populations. A baseline pretest cancer literacy survey consisting of 10 items was given to 349 participants, followed by the delivery of a cancer education presentation. Immediately following the presentation, participants were given a posttest with identical items to the pretest. Participants were primarily Caucasian (89.4%), female (68.7%), and in 10th through 12th grade (80.5%). Significant (*p* < 0.0001) increases in both average and median percent of correctly marked items were observed between the pretest and posttest (average, pretest = 56% versus posttest = 85%; median, pretest = 60% versus posttest = 90%). The scores for all individual items increased after the brief intervention. The results demonstrated a significant increase in cancer literacy levels immediately after the pilot educational intervention. We suggest that it may be possible to improve cancer literacy rates in Kentucky by integrating cancer education into middle and high school science and/or health education curricula. This could ultimately drive changes in behaviors that may help lower cancer incidence and mortality rates. Plans for future interventional studies measuring long-term cancer knowledge retention and resultant behavioral changes among middle and high school students as well as the feasibility of integrating cancer education into middle and high school curricula are also discussed.

**Electronic supplementary material:**

The online version of this article (10.1007/s13187-020-01696-3) contains supplementary material, which is available to authorized users.

## Introduction

Cancer is a leading public health problem in the United States (U.S.); there are over 1.7 million new cases each year and over 600,000 cancer deaths [1]. Although cancer is widespread and generally non-discriminatory, disparities in incidence and mortality exist across varying population groups, including residents of specific geographic regions. Notably, Kentucky ranks first in the nation in overall cancer incidence and mortality and experiences over 26,000 new cancer cases each year and over 10,000 cancer-related deaths [2]. Rural eastern Kentucky residents face some of the highest cancer incidence and mortality rates in the country [3, 4]. Residents of rural counties in Kentucky, specifically the Appalachian region, are 8% more likely to die from a preventable or screenable malignancy [5].

Cancer disparities in Kentucky are attributed to different factors, including elevated rates of inadequate exercise, poor diet, and smoking [4, 6–8]. Additionally, when compared to the national average, a higher percentage of Kentucky residents are at or below the federal poverty line, which greatly limits their access to health care [6]. The mountainous terrain of rural, eastern Kentucky and the region’s geographic isolation can make travel to the nearest preventive care facility, which may be several hours away, difficult [7]. Some residents may not have the time or the financial security to take a leave of absence from work to receive screenings or treatments in a facility far from home. Kentucky also struggles with low education levels, ranking 47th in the U.S. for educational attainment, serving as a barrier to health literacy [9].

Health literacy—the ability to understand health care information to make appropriate health decisions—is essential to taking the necessary precautions to protect oneself from health issues, yet one in three U.S. adults have limited health literacy [10–12]. Health literacy includes a general knowledge about the mechanisms of disease, possible treatments, and preventive measures. Health literacy has three dimensions [13]. The first, *functional literacy*, is measured based on an individual’s reading and writing skills that enable them to comprehend health information, such as basic facts on the biology of cancer. This is a surface level understanding of health literacy, as it does not take patient behavior into account. The second, *interactive health literacy*, includes how an individual is able to take an active role regarding their own health. Finally, *critical health literacy* is an individual’s ability to accept health-related advice and make appropriate decisions [13]. When considering cancer literacy in particular, it is important to take into account each dimension, as the ability of a patient to engage in proper screenings and treatment extends past functional health literacy [13]. Patients with low health literacy may have lower participation in cancer prevention activities, which may result in lower levels of cancer treatment and increased risk [14]. The desired outcome of increased cancer-related health literacy is that morbidity and mortality rates would decrease as patients begin to participate in preventive cancer behaviors [13].

Youth represent both a vulnerable population that are at risk of beginning harmful activities that can increase cancer risk (e.g., smoking, tanning) and a population that may be more amenable to cancer prevention and control interventions. These interventions include those associated with improving cancer literacy, which have the potential to lower cancer incidence and mortality rates [15–17]. With this in mind, the purpose of this pilot study was to establish connections with middle and high schools in Kentucky that would allow for the assessment of aspects of basic, functional cancer literacy in students prior to and immediately after participation in a brief cancer education intervention. Increasing cancer literacy among Kentucky’s youth could be an important long-term strategy for reducing cancer rates in the state.

## Methods

**Fig. 1:**
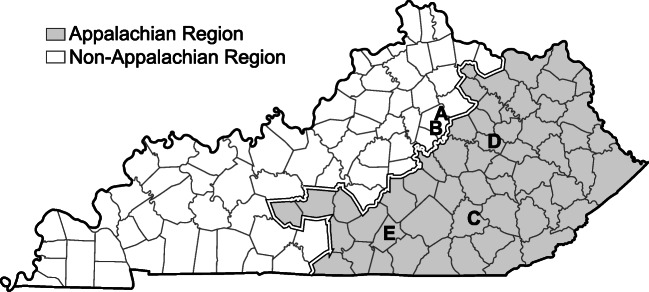
Map indicating the geographic location of each participating school

This pilot cancer literacy intervention occurred in participants’ schools during normal school hours, typically during a regularly scheduled science or health class. The target population was middle and high school students. Participants were recruited from four high schools and one middle school in Kentucky that chose to participate in the intervention; three of the high schools were located in the rural, Appalachian area of the state and the remaining schools were located in urban, central Kentucky (Fig. 1). Engagement with each school occurred through initial communication with individual science or health teachers or with school guidance counselors. The schools and participants were anonymized. General demographics, including gender, race, ethnicity, and grade level, were collected from each participant.

All pilot study procedures were approved by the University of Kentucky Institutional Review Board (Protocol 44637). Parental consent was waived. Student assent was obtained through engagement of the questionnaire after participants were informed of the study aims and methods and were assured that their identities would be anonymized.

Participants completed a paper-based demographic questionnaire and a 10-item pretest survey, observed a 30- to 45-min PowerPoint presentation (given by NLV), and then completed a 10-item posttest survey—identical to the pretest—immediately following the intervention. Participants had access to both the pre- and posttests during the duration of the intervention, and both tests were collected together following completion of the posttest. Because the intervention was given within a classroom setting, all students in attendance participated in the assessments and educational presentation. Given that all students who were present participated, the overall pre- and posttest response rate was 100%, but not every participant answered each question as they could skip questions.

The presentation topics included basic cancer biology principles, cancer risk factors, cancer statistics in the U.S. and Kentucky, and modifiable behaviors that can reduce the risk of cancer. The survey items were developed to test participants’ understanding of these topics; three of the questions (3, 6, and 7) were adopted from a previous study [18]. The demographic questionnaire and the pre/posttest are provided as supplemental material (Appendix 1).

One-way frequencies for all respondents were calculated for the demographic variables. The overall sample average and median percent of correctly marked items for both the pretest and posttest were calculated. A paired *t* test was used to test the null hypothesis that the difference between the average of the percent of correctly marked pretest and posttest items was equal to 0. The percent of correctly marked items was calculated for the entire sample and for demographic subgroups with similar hypothesis testing, along with 95% confidence intervals. Statistical analyses were performed in SAS 9.4 (Cary, NC).

## Results

**Fig. 2:**
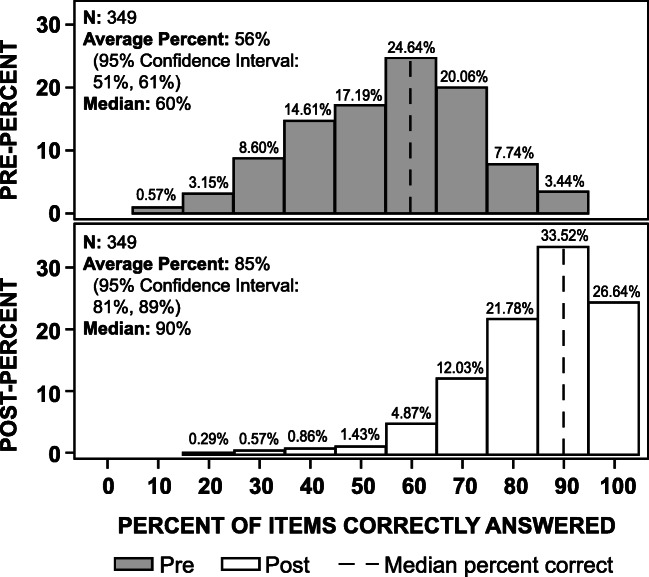
Overall pretest versus posttest scores on a 10-item cancer literacy survey**.** Participants (*N* = 349) were given a 10-item pretest before attending a 30- to 45-min cancer education presentation and afterwards participants completed a 10-item posttest that was identical to the pretest. The percent of items correctly answered were plotted

Participants (*N* = 349) were predominantly Caucasian (89.4%) and not of Hispanic or Latino descent (91.3%); these demographics closely match the overall demographics of Kentucky [19]. Over two-thirds of the participants were female (68.7%) and the majority (80.5%) were in 10th, 11th, or 12th grade (Table 1). The average percent of correctly marked items increased from 56% (95% confidence interval [CI], 51%, 61%) on the pretest to 85% (95% CI, 81%, 89%) on the posttest; median scores increased from 60% on the pretest to 90% on the posttest (Fig. 2).

**Table 1:** Participant characteristics

Demographic	*N*	%
School		
School A	24	6.9
School B	46	13.2
School C	154	44.1
School D	12	3.4
School E	113	32.4
Gender		
Female	239	68.7
Male	109	31.3
Race		
American Indian/Alaska Native	2	0.6
Asian	5	1.4
More than one race	30	8.6
White/Caucasian	311	89.4
Ethnicity		
Hispanic or Latino	30	8.7
Not Hispanic or Latino	317	91.3
Grade		
7	46	13.2
9	22	6.3
10	139	39.8
11	82	23.5
12	60	17.2

We observed a significant increase in the average percent of correctly marked items and percent responsiveness for each item from pretest to posttest. Item one (“What is cancer?”) had the lowest percent responsiveness (4.9%), indicating that the majority of participants were aware of this concept before the intervention. Item 9 (“How does Kentucky compare to other states in cancer rates?”) had the highest percent responsiveness (75.7%), indicating that participants were not aware of this concept before the intervention (Table 2). Items 1 and 5 were answered correctly by greater than 80% of participants on both the pretest and posttest, while items 1, 4, 5, 6, 7, and 8 were answered correctly by greater than 70% of participants on both the pre- and posttest, suggesting ceiling effects for these items (Table 2). There was a statistically significant (*p* < 0.0001) increase in the overall pretest versus posttest average and percent responsiveness scores for each school, gender, and grade level (Table 3).

**Table 2:** Cancer literacy survey items (correct answer in italics), pretest and posttest scores, and percent responsiveness

Question	*N*	Prescore (%)	Postscore (%)	% responsiveness	95% confidence interval (%)	*p* value
1. What is cancer? *a. Cancer is a disease caused by mutations that leads to uncontrolled cell growth.* b. Cancer is a virus that causes abnormal formations in the body. c. Cancer is a bacterial infection that causes abnormal processes in the body. d. Cancer is a metabolic disorder that causes changes in metabolism. e. Cancer is a mental disorder that causes changes in emotions.	346	93.4	98.3	4.9	2.6, 7.2	< .0001
2. What are the two major types of cancer? *a. Solid and liquid* b. Bone and organ c. Breast and lung d. Leukemia and metastatic e. All of the above	346	6.6	74.0	67.3	62.4, 72.3	< .0001
3. A benign tumor is cancerous. a. True *b. False*	331	62.5	87.9	25.4	20.7, 30.1	< .0001
4. What are common cancer risk factors? a. Age b. Carcinogens including environmental factors c. Obesity d. Viruses/infectious agents *d. All of the above*	343	78.7	97.1	18.4	14.2, 22.5	< .0001
5. What are some lifestyle choices that increase one’s likelihood of developing cancer? a. Smoking b. Unhealthy diet c. Risky behaviors *d. All of the above* e. None of the above	337	82.2	97.6	15.4	11.6, 19.3	< .0001
6. When cancer has metastasized is means it has: *a. Spread to other parts of the body* b. Spread to other parts of the originally affected organs c. Stopped spreading d. Been cured e. None of the above	330	70.6	92.7	22.1	17.6, 26.6	< .0001
7. A biopsy of a tumor is done to: a. Remove it *b. Diagnose it* c. Treat it d. Cure it e. None of the above	332	72.3	91.6	19.3	15.0, 23.5	< .0001
8. Cancer can impact populations or groups of people (for example, men versus women) differently? *a. True* b. False	343	77.6	97.7	20.1	15.9, 24.4	< .0001
9. How does Kentucky compare to other states in cancer rates? c. Kentucky is 15th in overall cancer incidence and mortality rates *d. Kentucky is 1st in the nation in overall cancer incidence and mortality rates* e. Kentucky has the lowest overall cancer incidence and mortality rates f. Kentucky has the same cancer incidence and mortality rates as other states g. None of the above	346	20.5	96.2	75.7	71.2, 80.3	< .0001
10. What four types of research are being conducted on cancer? a. Population/behavioral, transcriptional, clinical, systematic *b. Basic*, *clinical*, *translational*, *population/behavioral* c. Clinical, basic, qualitative, quantitative d. All of the above e. None of the above	327	11.6	41.3	29.7	24.7, 34.6	< .0001
Overall	349	55.8	84.9	29.1	27.4, 30.7	< .0001

**Table 3:** Pretest and posttest scores and percent responsiveness by school, gender, and grade for the 10-item cancer literacy survey

Demographic	*N*	Prescore (%)	Postscore (%)	% responsiveness	95% confidence interval (%)	*p* value
School						
School A	24	60.0	93.3	33.3	26.1, 40.6	< .0001
School B	46	50.7	80.7	30.0	26.4, 33.6	< .0001
School C	154	52.3	83.4	31.1	28.8, 33.4	< .0001
School D	12	55.8	84.2	28.3	20.8, 35.9	< .0001
School E	113	61.8	86.8	25.0	21.8, 28.3	< .0001
Gender						
Female	239	57.2	86.6	29.4	27.6, 31.3	< .0001
Male	109	52.8	81.2	28.3	25.1, 31.6	< .0001
Grade						
7	46	50.7	80.7	30.0	26.4, 33.6	< .0001
9	22	55.0	83.2	28.2	20.6, 35.8	< .0001
10	139	56.7	87.4	30.7	28.0, 33.4	< .0001
11	82	56.0	81.2	25.2	21.9, 28.6	< .0001
12	60	57.8	87.8	30.0	26.0, 34.0	< .0001

## Discussion

This pilot study established connections with schools, which allowed for an examination of the effects of a brief cancer-related educational intervention on cancer literacy levels among middle and high school students in Kentucky. The students were enrolled in schools that are geographically located in urban central and rural eastern Kentucky. There was a significant increase in the overall test scores following the pilot intervention. All items were responsive; there was a significant increase in individual test scores following the brief intervention. This indicates that participants’ cancer literacy increased, although the responsiveness was greater for some items. These data suggest that a brief educational intervention about cancer can increase middle and high school participants’ basic literacy of the disease.

Other studies have also demonstrated increases in cancer literacy knowledge levels as a result of educational interventions. A 2015 study of Mexican students’ knowledge of cervical and breast cancer used an educational strategy to increase clinical-focused cancer literacy; the results demonstrated a 21.2% increase in correct responses from pretest to posttest [20]. A 2018 study measuring health literacy in the context of cervical cancer screening in Japanese women found that an educational intervention increased health knowledge of the adult participants [21]. These studies point to the possibility of self-care improvements, including behavior changes that can lower cancer risk and increase how often patients seek care, alongside improved knowledge of a particular disease [19, 21]. The pilot intervention herein has similar potential.

This exploratory study should be interpreted cautiously and in context with its limitations. First, as a cross-sectional study of a convenience sample, the results may not be generalizable. It is difficult to know whether the results may be representative of all students in Kentucky or more broadly representative of students within the greater U.S., and, likewise, it is not clear whether these results could be generalized to adult populations. Second, participants had access to the pre- and posttest during the duration of the intervention and such access could have influenced their performance on the posttest. Third, the design of the study makes it difficult to determine the long-term educational effects of the intervention. Because the posttest was administered immediately after the intervention, it is impossible to discern from this pilot study whether the students retained the material or simply recalled it from their short-term memory. Lastly, several items were answered correctly by > 70% of the sample population on the pretest, suggesting a ceiling effect for these items, which limits the data range/variability. Although several items from our survey were validated in a previous study [18], the validity and reliability of our survey has not been confirmed. Despite the study’s limitations, this pilot work provides preliminary evidence that cancer literacy among youth may significantly increase even with a brief educational intervention.

Future studies will need to determine whether students retain the knowledge they obtain from any cancer education they receive. Based on the successful connections established with the five schools enrolled in this study, we have established connections with additional schools in Kentucky. Work is now underway to measure cancer knowledge retention several months after the brief intervention developed herein. We are also integrating additional measures that will determine whether participants change any behaviors over time as the result of the intervention. Lastly, we are also collecting data to understand the feasibility of incorporating cancer topics into science and/or health curricula at the newly participating schools.

## Conclusion

Cancer rates in Kentucky are elevated compared to general rates in the U.S. The use of educational interventions, especially among youth, could help increase cancer literacy. Such interventions can help students understand the basics of cancer, which could aid decision-making around modifiable cancer risk factors and health-seeking behaviors. As such, we recommend that school systems integrate evidence-based cancer education modules into their science or health education curricula.

## Electronic Supplementary Material

ESM 1 (DOCX 19 kb)
