# Supplementary material for: A Brief Educational Intervention Enhances Basic Cancer Literacy Among Kentucky Middle and High School Students
Source: J Cancer Educ. 2020 Jan 28;36(4):735–40. doi: 10.1007/s13187-020-01696-3 (PMC7388006; doi:10.1007/s13187-020-01696-3)
Supplement: Supplementary file 1 — (DOCX 19 kb) [file 13187_2020_1696_MOESM1_ESM.docx]

**Appendix 1**

**Participant characteristics**

1. Gender
   1. Female
   2. Male
2. Race
   1. American India/Alaska Native
   2. Asian
   3. Native Hawaiian or Other pacific Islander
   4. Black or African American
   5. White/Caucasian
   6. More Than One Race
3. Ethnicity
   1. Hispanic or Latino
   2. Not Hispanic or Latino
4. Grade
   1. 5^th^
   2. 6^th^
   3. 7^th^
   4. 8^th^
   5. 9^th^
   6. 10^th^
   7. 11^th^
   8. 12^th^

**Pretest**

1. What is cancer?
   1. Cancer is a disease caused by mutations that leads to uncontrolled cell growth.
   2. Cancer is a virus that causes abnormal formations in the body.
   3. Cancer is a bacterial infection that causes abnormal processes in the body.
   4. Cancer is a metabolic disorder that causes changes in metabolism.
   5. Cancer is a mental disorder that causes changes in emotions.
2. What are the two major types of cancer?
   1. Solid and Liquid
   2. Bone and organ
   3. Breast and Lung
   4. Leukemia and metastatic
   5. All of the above
3. A benign tumor is cancerous.
   1. True
   2. False
4. What are common cancer risk factors?
   1. Age
   2. Carcinogens including environmental factors
   3. Obesity
   4. Viruses/infectious agents
   5. All of the above
5. What are some lifestyle choices that increase one’s likelihood of developing cancer?
   1. Smoking
   2. Unhealthy diet
   3. Risky behaviors
   4. All of the above
   5. None of the above
6. When cancer has metastasized it means it has:
   1. Spread to other parts of the body
   2. Spread to other parts of the originally affected organs
   3. Stopped spreading
   4. Been cured
   5. None of the above
7. A biopsy of a tumor is done to:
   1. Remove it
   2. Diagnose it
   3. Treat it
   4. Cure it
   5. None of the above
8. Cancer can impact populations or groups of people (for example, men versus women) differently?
   1. True
   2. False
9. How does Kentucky compare to other states in cancer rates?
   1. Kentucky is 15^th^ in overall cancer incidence and mortality rates
   2. Kentucky is 1^st^ in the nation in overall cancer incidence and mortality rates
   3. Kentucky has the lowest overall cancer incidence and mortality rates
   4. Kentucky has the same cancer incidence and mortality rates as other states
   5. None of the above
10. What four types of research are being conducted on cancer?
    1. Population/Behavioral, Transcriptional, Clinical, Systematic
    2. Basic, Clinical, Translational, Population/Behavioral
    3. Clinical, Basic, Qualitative, Quantitative
    4. All of the above
    5. None of the above

**Posttest**

1. What is cancer?
   1. Cancer is a disease caused by mutations that leads to uncontrolled cell growth.
   2. Cancer is a virus that causes abnormal formations in the body.
   3. Cancer is a bacterial infection that causes abnormal processes in the body.
   4. Cancer is a metabolic disorder that causes changes in metabolism.
   5. Cancer is a mental disorder that causes changes in emotions.
2. What are the two major types of cancer?
   1. Solid and Liquid
   2. Bone and organ
   3. Breast and Lung
   4. Leukemia and metastatic
   5. All of the above
3. A benign tumor is cancerous.
   1. True
   2. False
4. What are common cancer risk factors?
   1. Age
   2. Carcinogens including environmental factors
   3. Obesity
   4. Viruses/infectious agents
   5. All of the above
5. What are some lifestyle choices that increase one’s likelihood of developing cancer?
   1. Smoking
   2. Unhealthy diet
   3. Risky behaviors
   4. All of the above
   5. None of the above
6. When cancer has metastasized it means it has:
   1. Spread to other parts of the body
   2. Spread to other parts of the originally affected organs
   3. Stopped spreading
   4. Been cured
   5. None of the above
7. A biopsy of a tumor is done to:
   1. Remove it
   2. Diagnose it
   3. Treat it
   4. Cure it
   5. None of the above
8. Cancer can impact populations or groups of people (for example, men versus women) differently?
   1. True
   2. False
9. How does Kentucky compare to other states in cancer rates?
   1. Kentucky is 15^th^ in overall cancer incidence and mortality rates
   2. Kentucky is 1^st^ in the nation in overall cancer incidence and mortality rates
   3. Kentucky has the lowest overall cancer incidence and mortality rates
   4. Kentucky has the same cancer incidence and mortality rates as other states
   5. None of the above
10. What four types of research are being conducted on cancer?
    1. Population/Behavioral, Transcriptional, Clinical, Systematic
    2. Basic, Clinical, Translational, Population/Behavioral
    3. Clinical, Basic, Qualitative, Quantitative
    4. All of the above
    5. None of the above
